# Supplementary material for: Cover cropping impacts on soil water and carbon in dryland cropping system
Source: PLoS One. 2023 Jun 5;18(6):e0286748. doi: 10.1371/journal.pone.0286748 (PMC10241367; doi:10.1371/journal.pone.0286748)
Supplement: Attachment — Submitted filename: suggestions 10.11.2022.docx [file pone.0286748.s001.docx]

suggestions:

Line 28: ...soil water was depleted by ....

Line 66: ... for increased water use efficiency ...

Line 119: ..., which can be 58-60% of SOM. (delete OC)

Line 239: three months ......

Line 239: Thus, the examined ....

Line 294: is it 20 or 40 tubes?

Line 314: subsamples per plot or per subplot? same for lines 335 and 336.

Lines 552-553: ... contents (TOC, POC and POXC) and clay content (Figure 6).

Lines 579-580: Results also showed that the surface layer 0-15cm stored soil water at wheat ...

Lines 592-593: ... ground cover, organic matter, stored soil water, soil quality, and health.

Lines 595-596: ANOVA test showed the ....

Lines 598-600: ... and increased yield by compared to ...?

Line 600: In this treatment TOC and POC changed by 7% ... (changed? increased or decreased?)

Line 601: ... soil biology as evidenced by an increase ...

Line 603 ... growing season of winter crop showed that managing summer cover ...

Line 604 ... during sensitive phenology stages ... (spelling)

Line 616 ... while enhancing chemical and biological ...

Line 624: ... soil water content at wheat sowing with the control, but enhancements ...

Line 625: ... the increase in yield and grain quality that was observed.

Line 641: However, mid and late-terminated plots ...

Line 688: delete 'could'

Line 724: spelling "concentration'

Line 753: delete 'had'

Line 758: ... affected soil water availability at wheat sowing time.

Lines 761-763: re-write for clarity.
