# Supplementary material for: Cover cropping impacts on soil water and carbon in dryland cropping system
Source: PLoS One. 2023 Jun 5;18(6):e0286748. doi: 10.1371/journal.pone.0286748 (PMC10241367; doi:10.1371/journal.pone.0286748)
Supplement: Attachment — Submitted filename: Response to Reviewers.docx [file pone.0286748.s002.docx]

**Response to Reviewers**

Manuscript ID: PONE-D-22-23600

*Cover cropping impacts on soil water and carbon in dryland cropping system*

Dear Editor and Reviewers,

Thank you so much for your valuable feedback and recommendations. Please see the response below as per your request.

**Journal Requirements:**

1. Please ensure that your manuscript meets PLOS ONE's style requirements, including those for file naming.

Author response: Thank you very much for the helpful comments. We have checked the style requirements and have ensured that all following the guideline of the journal. The following changes have been made:

-Author affiliation footnotes using numerical order, instead of alphabetical order

-1^st^ and 2^nd^ sets of equal contributors’ footnotes were added

-Figure and table captions used Bold type

-Figure citations changed from “Figure” to “Fig”

-Text font: Times New Roman

-Text size: 12

-Fig and table caption size: 11

-Table content size: 9

2. In your Methods section, please provide additional information regarding the permits you obtained for the work. Please ensure you have included the full name of the authority that approved the field site access and, if no permits were required, a brief statement explaining why.

Author response: Trials /experiments are part of the project BACI 2: (the Broadacre Cropping Initiative) funded by the Queensland government (Department of Agriculture and Fisheries) and the University of Southern Queensland, and conducted in a real farm. We have all the approvals to use the site for research. Property owner, property name and any related information cannot be disclosed for the protection of property confidentiality. The experimental trials were performed within the conditions of use of a currently authorised plant, fertiliser, and herbicide products.

A brief statement is added to” Section 2.1 Study site” as “Experimental trials are part of the project the Broadacre Cropping Initiative (BACI) funded by Queensland government (Department of Agriculture and Fisheries) and University of Southern Queensland. All the approvals have been obtained for conducting this research. Information such as property name and coordinates cannot be disclosed for confidentiality reasons.”

3. We note that Figure 1 in your submission contain [map/satellite] images which may be copyrighted. All PLOS content is published under the Creative Commons Attribution License (CC BY 4.0), which means that the manuscript, images, and Supporting Information files will be freely available online, and any third party is permitted to access, download, copy, distribute, and use these materials in any way, even commercially, with proper attribution. For these reasons, we cannot publish previously copyrighted maps or satellite images created using proprietary data, such as Google software (Google Maps, Street View, and Earth). For more information, see our copyright guidelines: <http://journals.plos.org/plosone/s/licenses-and-copyright>.

 We require you to either (1) present written permission from the copyright holder to publish these figures specifically under the CC BY 4.0 license, or (2) remove the figures from your submission

Author response: Thank you for your attention on copyright matters. The [map/satellite] in Fig 1 has now been replaced with a new map sourced from USGS National Map Viewer (public domain): <http://viewer.nationalmap.gov/viewer/>

Other photos in Fig 1 (c) were taken by first author.

4. Thank you for stating the following in the Acknowledgments Section of your manuscript:

“This work has been supported by the Cooperative Research Centre for High Performance Soils whose activities are funded by the Australian Government’s Cooperative Research Centre Program, along with support from both the Queensland Department of Agriculture and Fisheries and the University of Southern Queensland through the Broadacre Cropping Initiative.”

We note that you have provided funding information that is not currently declared in your Funding Statement. However, funding information should not appear in the Acknowledgments section or other areas of your manuscript. We will only publish funding information present in the Funding Statement section of the online submission form.

Please remove any funding-related text from the manuscript and let us know how you would like to update your Funding Statement. Currently, your Funding Statement reads as follows:

“AG received funding support provided by The Cooperative Research Centre for High Performance Soils (Project No. 2019/4_s.002

<https://soilcrc.com.au/>) and The Broadacre Cropping Initiative (<https://www.unisq.edu.au/research/institutes-centres/ilse/baci>).

The funders had no role in study design, data collection and analysis, decision to publish, or preparation of the manuscript.”

Please include your amended statements within your cover letter; we will change the online submission form on your behalf.

Author response: Thank you for this comment. Now all funding-related information has been declared in Funding Statement as “This work has been supported by the Cooperative Research Centre for High Performance Soils (Australian Government’s Cooperative Research Centre program), along with support from both the Queensland Government Department of Agriculture and Fisheries and the University of Southern Queensland through the Broadacre Cropping Initiative.”

In the Acknowledgements section, we revised the text as:

“This work has been supported by the Cooperative Research Centre for High Performance Soils (Australian Government’s Cooperative Research Centre program), along with support from both the Queensland Government Department of Agriculture and Fisheries and the University of Southern Queensland through the Broadacre Cropping Initiative.”

5. Review Comments to the Author

Reviewer #1: Cover cropping impacts on soil water and carbon in dryland cropping system is an interetsing study but if authors can suggest how much cover crop will help to incraese soil water and C in dryland cropping system it would be great as numbers ae missing.
Is terminations of summer cover crop practices is common practice ?
Recommenadtions seems not clear as its general please be specific.
Figure quality is also poor.

Author response: Thank you reviewer for this comment. It is not clear what is reviewer's definition of “how much cover cropping” is, this paper is about the effect of summer cover crop management i.e., termination time. It was in Line 625 -635 that “For the examined season, the early terminated summer cover crop was effective at increasing soil water at planting time by 2%, 4% and 1% in 0-15cm, 15-30cm and 30-45cm, and subsequently increased yield by 12% comparing to control. In this treatment TOC and POC increased by 7% and 9%, respectively (Table 4). Summer cover crop activated soil biology as evidenced by an increase in AMF sequence concentrations, in both A and B groups (Fig 3c and 3f). The soil water storage during the growing season of winter crop showed that managing summer cover crop could assist in providing enough water during sensitive phenology stages (Fig 5). Although there was an underlying correlation detected between soil water, biomass, yield (Fig 6), the combined effect from changes in soil water and OC increased yield (12%) and quality i.e., protein (5%) in early terminated cover crop plots (Table 4).”

The sections 3.1, 3.2 and 3.4, along with Table 5 presented all details as summarised above.

Yes, termination of the summer cover crop is a common practice in the study region because 1) provides ground cover during fallow and adds organic materials into the soil; 2) prevents excessive soil moisture loss due to plant water use by cover crop; 3) to avoid/decrease potential interference with the planting operation of the following cash crop (i.e. winter wheat in our case).

It was in the introduction Lines 101-103 “Cover crops under dryland conditions are more likely to compete for soil water and nutrient resources with the cash crop [15,32], so appropriate termination time becomes crucial for cover crop management to avoid the competition [34]. …..”

Now the paper is revised in Lines 101-112:

“Cover crops under dryland conditions are more likely to compete for soil water and nutrient resources with the cash crop [15,32], therefore appropriate termination time becomes crucial for cover crop management to avoid the competition, particularly in water-limited regions where soil nutrients and water-use efficiency are often low [34, 35]. By timely terminating cover crop in consideration of factors like cover crop growth stage and planting time of the cash crop, it is helpful to prevent soil water loss from evapotranspiration and can consequently avoid depletion of available soil water at sowing time of the cash crop [36]. In dryland cropping systems or under drought conditions, cover cropping with optimised termination timing is a common practice and has been reported to increase soil water to avoid water depletion [37, 38].”

Thank you again for the comment on the recommendations. In *Section 4. Limitations and recommendations*, we have now revised our recommendations as the following text in lines 837-848:

“Therefore, it is recommended that summer cover crop incorporation could help to promote soil health (organic carbon accumulation and microbial activities) through residue retention. This study also showed the importance of the timing of cover crop termination, for its impact on soil water storage. For future studies, , it is important to consider a number of factors prior to implementing cover crop practices: 1) decision on planting and termination of cover crops should be carefully planned; 2) considering the impacts of cover cropping because it may not necessarily achieve all the benefits (adequate soil water preservation, yield increase, , carbon accumulation, microbial health enhancement) that can be affected by many factors such as soil condition, growing season, climate, management and investment decisions; 3) considerint the potential impact of cover cropping on soil nitrogen retention and their regulation effects on nitrogen cycling processes. .”

Figures were all submitted in TIF format with a quality of 1500dpi. We will resubmit all figures including revised Fig6 in the same format suggested by the guideline. Please download the original size to access them.

Reviewer #2: Zhang et al. investigated the effects of replacing conventional fallow period with summer sorghum cover crop and its termination timing in SW QLD on soil water and carbon dynamics, AMF abundance and subsequent winter wheat. The manuscript indicates early termination of summer cover crop improve AMF, soil water accumulation at wheat planting, and wheat grain yield, % protein and WUE. It is quite interesting to figure out how cover cropping can affect soil C cycling, soil water balance and subsequent crop yield in dryland environments. The topic of this manuscript is of interest of the readership of PlosOne, as well as agricultural community at large. However, there are several flaws need to be addressed before the acceptance of the manuscript.
My main comments are:
• The manuscript presented a result of one-year field studies in one location with 5 reps. One of the key features of the dryland cropping system in the SW QLD is high climatic variability (see your statement in line 224 on the extreme weather variability of this region). This study is limited to single year and location and did not capture at least two years of this variability.

In addition, while the 5 replications of the experimental treatments could provide an estimate of the variability around the measurements, the authors did not present any measure of error (e.g standard error) around the estimate of the mean (see Fig 3-5, Table 6 for example) of the measured variables. This limits the reproducibility or the relevance of the findings from this manuscript.

Author response: We appreciate Reviewer#2’s feedback.

This paper is part of Author#1’s PhD study. Considering the timeframe and budget provided for this PhD research, it was impossible to conduct field experiments and data collection beyond 1 year. To handle this constraint, we included 5 replications for each treatment. However, should consider that in practice, farmers apply crop rotations across a sequence of growing seasons, which means the management and crop sequence will change in the following year. Our focus is to investigate the changes in soil water and organic carbon under 1-year cover crop incorporation, in a year that has a typical rainfall of the region.

In terms of rainfall, as the main driver of soil water storage and crop productivity in a dryland system, for the examined year (2021), we have compared the average rainfall to average rainfall in the period of 1990-2020 (also please see Fig 1). Therefore, as was stated in the manuscript L258-259 “the examined year represented seasonal condition in an area that winter crop yield is highly dependent on soil water storage”.

Thank you reviewer for the comments about the measure of error. Fig 3 and Table 6 have been revised with the addition of standard errors. Fig 4 and 5 present the mean soil water content of each treatment over summer and winter seasons and it would be very messy to show standard error in the figure. Therefore, we are happy to provide a supplementary material for the soil water data if needed.

• The introduction and the results are very extensive, and the discussion is more general, without going into the effects on the soil that caused a greater or lesser use of N and consequently of the use of water (accumulation of C, nitrification, denitrification, immobilization, etc.), such as for example activity of microorganisms, and their effect on N-mineral transformation in soil? Thus, I believe that some changes should be made to provide a more focused discussion.

Author response: Thank you for pointing this out. The cover crop practice can impact soil N cycles and its interactions with other soil processes, however, it is beyond the scope of this paper as we explore the impact of cover cropping on soil water, soil organic carbon and subsequent crop yield. The paper is already a long paper, we are exploring N in another publication. However, we appreciate the reviewer’s comment and we have added some changes to the recommendation section as stated in Line 845-846:

“3) the potential impact of cover cropping on soil nitrogen retention and their regulation effects on nitrogen cycling processes.”

• The text in general is very speculative, with a long, repetitive introduction, the material and methods, it needs more information about the herbicide spray, definitions of fertilizers used in the cultures. For e.g what is the composition of the Starter Z (40kg/ha)?

Author response: Thank you reviewer for the comment on the introduction. We have followed journal guideline for writing this section. The reviewer has not provided clear comments to address, however, now we have edited and shortened the introduction.

Now also the paper is revised by modification of Table 1 and added:

Herbicide application: Roundup UltraMax 0.2 ml/ha applied at each termination (Active ingredient 570g/L Glyphosate)

Fertiliser application: Starter Z 25kg/ha on 15/01/2021 and 40kg/ha on 28/05/2021 (Mono ammonium phosphate plus zinc, containing 10% N, 22% P, 2% S and 1% Zn)

• The authors tried a lot, but the way of personation is still poor. Avoid we, I, you, they, etc. in the manuscript.

Author response: Thank you for this. Although nowadays is common to use such a language, now the paper is revised by reducing occurrences of using “we” and “they” to twice each.

• There are extensive citations in the manuscript. In this sense, the manuscript provides a lot of information, but with data that do not add much information relevant to the proposed theme. Overall, the article must be improved with more clarity and a set of information with scientific justification.

Author response:

We concur with the reviewer that this manuscript used a lot of citations. However, this paper covers a range of information that is related to the impact of cover crop practice on soil moisture, soil carbon, AMF and subsequent crop yield, which led to the reason why we measured all these related soil attributes, and tried to relate our results with existing studies/literature. Therefore, the citations used in Introduction and Discussion were carefully considered and chosen to support each statement.

• The results and discussion are too long and unwieldy. There is need to streamline it based on the hypothesis of the paper. Many of the figures are repetitive.

Author response:

Thank you for the comment. We accept that there is an unavoidable complexity in this work as we had to handle a large amount of data.

**Fig 1** presents the Case study site (a) approximate location of trial sites, (b) monthly average rainfall of the season in comparison with the long-term average, (c) soil surface at the trial site

**Fig 2** presents Use of the neutron moisture meter probe in the field.

**Fig 3** presents (a) Soil total organic carbon (TOC) and particulate organic carbon (POC) and permanganate oxidizable carbon (POXC) at 10 cm depth at the time of termination of summer cover crop; (b) TOC and POC measured at harvest time of winter wheat; (c) volumetric soil water (VSW) content at 15cm, 25cm and 35cm and (d) whole profile soil water at wheat sowing time; AMF Group A (e) and Group B (f) measured at the time of termination of summer cover crop; (g) wheat above-ground dry biomass measured at grain filling and early maturity phenology stages and yield at harvest; (h) grain quality. SC: Summer control; ET: Early termination; MT: Mid termination; LT: Late termination.

**Fig 4** presenting Fig 4. Soil water storage of top layers (0-15cm, 15-30cm and 30-45cm) and whole profile (0-150 cm) during summer cover crop. At the planting time, in the absence of rainfall for 18 days before planting, Early termination had stored higher water in surface soil (0-30cm) while performing the same as fallow for all other layers and averaged for the whole profile.

**Fig 5** presenting Soil water in topsoil layers (0-15cm, 15-30cm and 30-45cm) and the whole profile (0-150cm) during the winter season.

**Fig 6** presenting Fig 6. The two-dimensional principal subspace for the observed data. SW: Soil water; BD: Bulk density.

**Fig 7** presents the relationship between total organic carbon and particulate organic carbon (POC) and permanganate oxidizable carbon (POXC). Top left: TOC and POC in summer and winter control plots; Bottom Left: TOC and POXC measured in summer control plots; Top right: TOC and POC in summer and winter cover crop plots; Bottom right: POXC and TOC in summer cover crop plots. The grey shadow area represents a 95% confidence interval.

**Fig 8** presents Relationship between soil water at the top layers and whole profile at planting time and wheat yield and biomass accumulation at grain filling and early maturity stages. The grey shadow area represents a 95% confidence interval.

As explained in above, We do not have overlapping presentations in the Figs and the reviewer didn’t specify any. This study aimed to address the research objectives that were stated in Line 214-220 “The objectives of this study were to evaluate the effect of summer cover crop practices on 1) soil OC (i.e., TOC, POC and POXC) and soil AMF DNA sequence concentrations at termination time of summer cover crop; 2) soil water across the soil profile (i.e. 0-150 cm) over the growing season and at the sowing time of the following cash crop; and 3) investigate the dependencies between soil OC, soil water at planting, wheat biomass, yield and grain quality.”

However, we have now revised the results sections to align with the research objectives, the revised section layout please see below:

“3.1 Soil organic carbon affected by cover cropping”

“3.2 Soil water storage affected by cover cropping”

“3.2.1 Changes in soil water storage over summer”

“3.2.2 Changes in soil water storage over winter cover crop”

“3.3 Arbuscular Mycorrhizal Fungi affected by cover cropping”

“3.4 Wheat biomass, yield and grain quality”

“3.5 Relationships among the variables”

“3.6 TOC and Labile OC relationships”

“3.7 Yield affected by soil water at wheat planting”

**Some line-by-line recommendations**

Author response: Thank you so much for the recommendations. All line-by-line recommendations have been addressed and revised in the manuscript. Please see below for each addressed change:

Note: As lots of revision and changes were made to the manuscript, some of line numbers are now shifted.

L17-18: The practice cover cropping has a limited application to some part of the globe (e.g arid areas or even some areas with unimodal rainfall pattern). May be change to “many part of the globe”

L17-18 (now L19-20) is now revised as “Incorporating cover crops into rotation is a practice applied across **many parts of** the globe to enhance soil biological activities.”

L20: change objectives to ‘objective”

L20 (now L22-23) is now revised as “The **objective** of this study **was** to evaluate…”

L21: change “different terminations of summer cover crop practices” to “different timing of summer sorghum cover crop termination”

L21 (now L23-24) is now revised as “… to evaluate the effect of **different timing of summer sorghum cover crop termination** on soil water…”

L22: I didn’t see results of interaction. Perhaps it was a mediating effect

L22 (now L25-26) is revised as “…their ~~interactions~~ **mediating effects on wheat yield.**”

L23: remove “planted after a summer sorghum cover crop”

L23 (now L26): “planted after a summer sorghum cover crop” **removed**.

L28-29: “Under late terminated summer cover crop, soil water depleted by 7% at wheat 29 planting which resulted in 61% decline in yield” to ‘Under late terminated summer cover crop, there was 7% soil water depletion at wheat planting which resulted in 61% decline in yield”

L28-29 (now L31-32) has now revised as “**Under late terminated summer cover crop, there was 7% soil water depletion at wheat planting which resulted in 61% decline in yield**.”

L37: soil water retention is different from soil water storage. Paraphrase.

L37 (now L42) has been revised as “…**soil water storage** during season…”

L66: “for increased water used efficiency” or “for increased water use efficiency”

L66 (now L70) has corrected the spelling error “used” to “**use**”.

L66: I think the contention that cover cropping has been adopted across the globe is not factually true.

L66 (now L71) has been revised as “Among these, the use of cover crops has been adopted across **many parts of** the globe to manage both soil water and nutrients…”

L143: Check GRSP

L143 (now L157-158) has revised the parentheses in “~~(~~glomalin-related soil proteins~~)~~ (GRSP)”

L164-168: Extensive studies- only 1 cited reference

L164-168 (now L179-180) has added references and removed a repeated sentence in L177-178, now as “Extensive studies **[75,76,77]** have been conducted to investigate the response of plant available water capacity (PAWC) to variation of soil OC or SOM content.”

L179: check “[80] reviewed that an increase”

L179 (now L195) has revised as “**Study [80] showed** that…”

L226: “2%” hanging

There is no “2%” in L226.

L279: 5.0 cm height of …

L279 (now L302-303) now revised as “4.3 cm diameter by 5.0 cm ~~height~~”

L280: remove “height”

L280 (now L303) **removed “height”.**

L290-294: Describe what was done not what ought to be done

L290-294 (now L315-320): Thank you reviewer for this comment. It is important to mention the principle of NMM approach, however, we have now revised the sentences as “**Soil water monitoring using NMM approach** was based on the physical interaction of radioactive neutrons with hydrogen atoms, and it had better control of both bulk density and hydrogen atoms during the calibration process [106]. **On the basis of the** positive relationship between the relative neutron count rate and volumetric soil water, **soil water content was estimated** from the NMM readings (Fig 2).”

L332: What does the phrase “soil microbial health” means? Perhaps “soil health”

L332 (now L361) has been removed.

L412: fallow (control) or control… use a consistent phrase for the control treatment.

L412 (now L445) is now using “**control**” for the control treatment.

L420-421: This did not make any sense “There was little difference between control and early termination difference varied across the profile (Figure 3)”

L420-421 (now L463-464): “There was little difference between control and early termination difference varied across the profile (Figure 3)” **removed**.

L440-441: This sentence is not necessary” Following the summer cover crop, wheat was planted in plots that were under previous 441 treatments, here we call plots with their treatment name during summer”

L440-441 (now L540-541): the sentence is now **removed**.

L593: The phrase “microbial health” did not make any sense

L593 (now L686-702) is now revised as “Incorporation of cover crops has been practised as a means to manage ground cover, organic matter, stored soil water, soil quality and health.”

L952-608: This repetition of the previous section

L592-608 (now L686-702) is a summary of the results section, not a repetition.

Reviewer #3: suggestions:
Line 28: ...soil water was depleted by ....

Thank you Reveiwer#3 for your line-by-line suggestions.

L28 (now L31-32) is revised as “Under late terminated summer cover crop, there was 7% soil water depletion at wheat planting which resulted in 61% decline in yield”.

Line 66: ... for increased water use efficiency ...

Line 66 (now L70): spelling error is now corrected as “for increased water **use** efficiency”.

Line 119: ..., which can be 58-60% of SOM. (delete OC)

L119 (now L133) is now revised as “which can be 58-60% of SOM”.

Line 239: three months ......

L239 (now L260) added “three” in “…which rainfall distribution over **three** months…”.

Line 239: Thus, the examined ....

L239 (now L263) added “the” in “Thus, **the** examined year is ….”.

Line 294: is it 20 or 40 tubes?

L294 (now 324) is now revised as “**40** tubes were reinstalled….”

Line 314: subsamples per plot or per subplot? same for lines 335 and 336.

Line 314 (L344-345): Thank you reviewer for pointing this out. For OC sampling at the termination of summer cover crop, it was “10 subsamples per plot”. For OC sampling at the end of the winter trial, it was “10 subsamples for subplot”. Therefore, the sentence “**At the end of winter trial, soil samples were sampled for TOC and POC contents (10 subsamples for subplot).**” Has been added to L337-338.

Lines 552-553: ... contents (TOC, POC and POXC) and clay content (Figure 6).

L552-553 (now L645646) is revised as “…soil OC contents (TOC, POC and POXC) and **clay** content (Fig 6)”.

Lines 579-580: Results also showed that the surface layer 0-15cm stored soil water at wheat ...

L579-580 (now L673-674) is now revised as “Results also showed that **the** surface layer 0-15cm **stored** soil water at wheat sowing…”.

Lines 592-593: ... ground cover, organic matter, stored soil water, soil quality, and health.

L592-593 (now L686-687) is now revised as “Incorporation of cover crops has been practised as a means to manage ground cover, organic matter, stored soil water, soil quality and health.”

Lines 595-596: ANOVA test showed the ....

L595-596 (now L689) is revised tense, now as “ANOVA test **showed**…”.

Lines 598-600: ... and increased yield by compared to ...?

L598-600 (now L692-694) is now revised as “…increased yield by 12% **compared to control**”.

Line 600: In this treatment TOC and POC changed by 7% ... (changed? increased or decreased?)

L600 (now L696) is revised “changed” to “**increased**”.

Line 601: ... soil biology as evidenced by an increase ...

L601 (now L697) is now revised as “Summer cover crop activated soil biology **as evidenced** by…”.

Line 603 ... growing season of winter crop showed that managing summer cover ...

L603 (now L698-699) is now revised as “The soil water **storage** during the growing season of winter crop **showed** that…”

Line 604 ... during sensitive phenology stages ... (spelling)

L604 (now L700): spelling of “**phenology**” is now corrected.

Line 616 ... while enhancing chemical and biological ...

L616 (now L711): added “chemical and”, now revised as “…while enhancing **chemical and** biological indicators…”

Line 624: ... soil water content at wheat sowing with the control, but enhancements ...

L624 (now 720) is now revised as “…soil water content at wheat sowing **with** the control…”

Line 625: ... the increase in yield and grain quality that was observed.

L625 (now L721-722) is now revised as “…played a role in the increase in yield and grain quality **that was** **observed**.”

Line 641: However, mid and late-terminated plots ...

L641 (now L737) is now revised as “However, mid and late**-terminated** plots…”.

Line 688: delete 'could'

L688 (now L787) is now revised as “…an increase in POC content **contributed** to…”.

Line 724: spelling "concentration'

L724 (now L823): spelling is now corrected as “**concentration**”.

Line 753: delete 'had'

L753 (now L869): “had” is **deleted**.

Line 758: ... affected soil water availability at wheat sowing time.

L758 (now L874) is now revised as “…affected soil water availability at wheat sowing **time**.”.

Lines 761-763: re-write for clarity.

L761-763 (now L877-881) is now rewritten as “There was a 4% increase in **surface** soil water at winter wheat sowing **time** under optimum summer cover crop, but effects were not proportional in yield increase i.e., 12% which **suggests that yield increase could be benefited** from enhancement in soil health i.e., soil **OC** and **potentially microbial activities**.”.
