# Supplementary material for: Cover cropping impacts on soil water and carbon in dryland cropping system
Source: PLoS One. 2023 Jun 5;18(6):e0286748. doi: 10.1371/journal.pone.0286748 (PMC10241367; doi:10.1371/journal.pone.0286748)
Supplement: Attachment — Submitted filename: Response to Reviewers.docx [file pone.0286748.s003.docx]

**Response to Reviewers**

Manuscript ID: PONE-D-22-23600R1

*Cover cropping impacts on soil water and carbon in dryland cropping system*

PLOS ONE

Dear Editor and Reviewers,

Thank you so much for your further valuable comments. Please see the response below as per your request.

**Journal Requirements:**

Please review your reference list to ensure that it is complete and correct. If you have cited papers that have been retracted, please include the rationale for doing so in the manuscript text, or remove these references and replace them with relevant current references. Any changes to the reference list should be mentioned in the rebuttal letter that accompanies your revised manuscript. If you need to cite a retracted article, indicate the article’s retracted status in the References list and also include a citation and full reference for the retraction notice.

All references in manuscript have been checked and revised in EndNote to meet Plos One Reference Requirement-Vancouver Style.

**Reviewer Comments to the Author:**

Reviewer #2: The author has addressed most of the comments raised. Overall, I commended the authors for addressing the key issues. However, I still think the text is too long particularly the introduction section. The authors indicated that because they have looked at different aspects of the cover crop effects, the text has to be this long. This is research manuscript not a review article. However, the same information can be presented with shorter introduction of 5-6 paragraphs. The introduction reported of several long-term cover crop studies on soil water, SOC and indicators of soil health, and readers might think this study will also be a long-term experiment. I recommend the introduction to be shorten, by providing the background on the study, the existing knowledge gap(s) on the subject and the need for the reported study and finally a brief summary of the work and its significance. A total of 94 citations was included in the introduction, and as such the text is unwieldy.

Thank you so much Mr. Garba for your feedback and recommendations. Introduction section is now revised and shortened to provide more clear presentation. The revised introduction part is now structured as below:

*Paragraph 1*: Importance of soil water in dryland cropping systems. Impact of fallow replacement with cover crops to manage soil water and soil health. (Background on the study🡪managing soil water in rainfed cropping system by replacing fallow with cover crops)

*Paragraph 2*: How cover crop practice affects soil water and soil organic carbon, and their interactions with each other.

*Paragraph 3*: Role of arbuscular mycorrhizal fungi (AMF) in cropping system and the effect of cover crops on AMF and their interactions with soil water and carbon availability.

(*Paragraph 2-3*: Existing knowledge)

*Paragraph 4*: Research gap and the need for the reported study, which can be significant with a dryland cropping system in Australia (State of Qld in particular). Research objectives.

Paragraph 5: A brief summary of the work and its significance.

Total number of references used in this section has been reduced to 74.

**Other minor line by line recommendations:**

24: Change "on-farm trials" to "on-farm trial" as it is only one trial.

Line 24: “on-farm trials” has been revised as “**on-farm trial**”.

30: late termination or "late terminated cover crop" Be consistent as you used "early/late terminated" before.

Line 30: “late termination” has been revised as “**late terminated cover crop**”.

86: reduce rather than "prevent" soil water loss from evapotranspiration. The cover crop must use water even if terminated early.

Line 86 (now Line 78): Word “prevent” has been changed to “**reduce**”.

104: Soil organic material or soil organic matter?

Line 104 (now Line 85): “soil organic material” has been changed to “**soil organic matter**”.

236: Trial site not sites

Line 236 (Now Line 184): “Trial sites” in Fig 1 caption has been revised as “**Trial site**”.

386-514: Should how the % relative change be described in the data analysis sub-section?

Thank you reviewer for pointing this out. The % relative change in the data analysis sub-section ( is referred to the comparison with the control. Fig 3 shows the comparison of the soil characteristics between treatments and the control.

448-449: soil water became similar to other treatments at the flowering stage... How soil become similar to other treatments? Perhaps not significantly different?

Line 448-449 (now Line 396-397): “…soil water became similar to other…” has been revised as “…soil water **was not significantly different** to other…”

567: Incorporation of cover crops into what?

Line 567 (now Line 515): “Incorporation of cover crops” has been revised as “**Incorporation of cover cropping into a crop-fallow system**”.

569-583: This is already presented results not discussion. Can be remove.

This section is an important introduction part for the discussion section, which should not be removed. Now it has been **revised** as below**:**

“…For the examined season, the early termination of summer cover crop resulted in a 2%, 4% and 1% increase in soil water at planting time at depths of 0–15cm, 15–30cm and 30–45cm, respectively, and subsequently led to a 12% increase in wheat compared to the control. Additionally, this treatment increased TOC and POC levels by 7% and 9%, respectively (Table 4). The summer cover crop was found to enhance soil biology as evidenced by an increase in AMF sequence concentrations in both A and B groups (Figure 3c and 3f). Managing summer cover crop effectively could potentially increase soil water storage during the growing season of the winter cash crop, which could be crucial for sensitive phenology stages (Figure 5). While an underlying correlation was observed between soil water, biomass and yield (Figure 6), the combined effect of changes in soil water and organic carbon resulted in an increased yield (12%) and improved quality, such as a 5% increase in grain protein (5%) in the early-terminated cover crop treatment (Table 4). “

614-619: did the residue affect wheat emergence in the current study? No results on establishment count?

The summer cover crop residue was likely affected the winter wheat emergence in this study based on the wheat biomass measurement. We did not collect data for the plant establishment count. This is a good point, and we will consider it for our future trial study. Thank you so much for pointing this out.

726: POC or POXC?

Line 726 (now Line 661): **POC**. No change was made.

Reviewer #3: This study shows well how cover crop terminating timing plays an important role in managing soil water storage and yields under dryland cropping systems. The study also tracks how chemical and biological indicators (TOC, POC, AMF sequence concentration, and microbial activities) tie up together in enhancing soil health. This study is particularly important in that it highlights the importance of cover crops under dryland conditions in semi-arid areas, where generally farmers are skeptical about cover crop adoption based on the idea of saving moisture for the main crop. Well-managed cover crops in semi-arid dryland are potentially more beneficial than elsewhere where precipitation is in abundance.

Thank you Dr. Mubvumba for your kind words and your comments. We would like to emphasize that the research gap is lack of quantified interactions of those soil characterises, which was address by this study.
